# Evaluation of the inflammatory response to *Kudoa septempunctata* genotype ST3 isolated from olive flounder (*Paralichthys olivaceus*) in Caco-2 cells

**DOI:** 10.1051/parasite/2018013

**Published:** 2018-03-13

**Authors:** Meejung Ahn, Hyun Ju Ko, Jeongtae Kim, Yeounghwan Jang, Taekyun Shin

**Affiliations:** 1 College of Veterinary Medicine and Veterinary Medical Research Institute, Jeju National University, Jeju 63243 Republic of Korea; 2 It’s Hanbul Co., Ltd. Research Institute 4, Nonhyeon-dong 249, Gangnam-gu, Seoul 06101 Republic of Korea; 3 Ocean and Fisheries Research Institute, Jeju Special Self-Governing Province, Pyoseon-myeon, Segwipo-si, Jeju 63629 Republic of Korea

**Keywords:** Caco-2 cells, genotype ST3, *Kudoa septempunctata*, *Paralichthys olivaceus*

## Abstract

*Kudoa septempunctata* (Myxosporea, Multivalvulida) is a parasite of the trunk muscle of cultured olive flounder (*Paralichthys olivaceus*). We investigated whether *K*. *septempunctata* genotype ST3 spores induce cell damage and the secretion of inflammatory mediators in Caco-2 cells, which exhibit characteristics similar to human intestinal epithelial cells. Purified *K*. *septempunctata* spores were heated at 95 °C for 5 min. Lactate dehydrogenase (LDH) release was measured to determine the efficacy of denaturation. Naïve and heated spores, lipopolysaccharide (positive control) and vehicle (negative control) were added to Caco-2 cells. Cells were subjected to the cytotoxic LDH assay and western blot analysis to examine the expression of inducible nitric oxide synthase (iNOS) and cyclooxygenase (COX)-2. Supernatants were collected to measure nitric oxide (NO) and prostaglandin E2 (PGE_2_). Most spores were denaturated by heating, and the spore morphology was found to be wrinkled with shell valves and polar capsules. In addition, cytotoxicity and inflammatory mediators, such as NO, PGE_2_, iNOS, and COX-2, remained unchanged in Caco-2 cells following exposure to naïve and heated spores compared with the positive controls. Collectively, the findings of this study imply that spores of *K*.* septempunctata* genotype ST3 do not cause inflammation in Caco-2 cells.

## Abstract (fr)

*Kudoa septempunctata* (Myxosporea, Multivalvulida) est un parasite des muscles du cardeau hirame cultivé (*Paralichthys olivaceus*). Nous avons étudié si les spores du génotype ST3 de *K. septempunctata* induisent des lésions cellulaires et la sécrétion de médiateurs inflammatoires dans les cellules Caco-2, qui présentent des caractéristiques similaires aux cellules épithéliales intestinales humaines. Les spores de *K. septempunctata* purifiées ont été chauffées à 95 ° C pendant 5 min. La libération de lactate déshydrogénase (LDH) a été mesurée pour déterminer l’efficacité de la dénaturation. Des spores naïves et chauffées, ainsi que des lipopolysaccharides (témoins positifs) et un véhicule (témoin négatif) ont été ajoutés aux cellules Caco-2. Les cellules ont été soumises à l’analyse de la LDH cytotoxique et à un western blot pour examiner l’expression de l’oxyde nitrique synthase inductible (iNOS) et de la cyclooxygénase (COX)-2. Les surnageants ont été recueillis pour mesurer l’oxyde nitrique (NO) et la prostaglandine E2 (PGE2). La plupart des spores étaient dénaturées par le chauffage, et la morphologie des spores était ridée avec des valves de coquille et des capsules polaires. En outre, la cytotoxicité et les médiateurs inflammatoires, tels que NO, PGE2, iNOS et COX-2, sont restés inchangés dans les cellules Caco-2 après exposition à des spores naïves et chauffées par rapport aux témoins positifs. Collectivement, les résultats de cette étude impliquent que les spores du génotype ST3 de *K. septempunctata* ne provoquent pas d’inflammation dans les cellules Caco-2.

## Introduction

*Kudoa* *septempunctata* (Myxosporea: Multivalvulida) has been identified in the trunk muscle of cultured olive flounder (*Paralichthys olivaceus*), occasionally causing illness due to food poisoning in Japan [12,15]. To prevent food poisoning caused by *K*. *septempunctata*, the Ministry of Health, Labor, and Welfare of Japan recommends freezing at −15 °C to −20 °C for more than 4 h or heating at 75 °C for more than 5 min. *K*. *septempunctata* spores are composed of six or seven shell valves and polar capsules [15], which are genetically classified into three groups; both ST1 and ST2 genotypes are commonly found in Japan, while ST3 is dominant in the Republic of Korea [19]. Oral administration of *K*.* septempunctata* spore*-*infected olive flounder is strongly believed to be associated with a diarrhea outbreak in ddY suckling mice [12]. However, the effect of isolated *K*. *septempunctata* spores in mammals was evaluated in adult BALB/c and suckling ddY mice fed spores of *K*. *septempunctata* genotype ST3, which caused no pathological change in the gastrointestinal tract despite detection of the gene in the feces of infected mice [1,9].

Some species of protozoa, such as *Giardia lamblia*, *Toxoplasma gondii*, *Cryptosporidium parvum*, and *Entamoeba histolytica*, cause infection-associated diarrhea due to intestinal invasion and reproduction in the intestine [4,14]. These protozoan infections cause inflammatory responses between cells in the gut, which results in the secretion of inflammatory mediators, such as tumor necrosis factor (TNF), interleukin (IL)-1, IL-6, nitric oxide (NO), soluble compounds, and eicosanoids, and cause the host immune system to become infected with protozoan parasites [13,18]. However, *K*. *septempunctata* genotype ST3-infected olive flounder showed no difference in the expression of immune-related genes, including ILs, cluster of differentiation 40, FAS ligand, TNF receptor-1, interferon regulatory factors, NOD-like receptor CARD domain 5, Toll-like receptors, and complement C3, between uninfected and infected flounder fish [10]. Although the *K*. *septempunctata* ST1 and/or ST2 sporoplasm has been reported to invade human epithelial cells to terminate monolayer confluence and contribute to diarrhea associated with infection [16], few studies have investigated the mechanism by which the expression of inflammatory molecules by these spores contributes to diarrhea *in vitro*.

In this study, we investigated whether these spores induce cell damage and the secretion of inflammatory mediators in Caco-2 cells, which exhibit characteristics similar to human intestinal epithelial cells, to examine the mechanisms by which *K*. *septempunctata* genotype ST3 spores cause inflammation.

## Materials and methods

### Spore preparation

*K*. *septempunctata-*infected olive flounder samples were collected from an aquaculture fish farm in Jeju Island. These fish were periodically screened by microscopic examination of crude suspensions of muscle tissue at 400 × magnification for the presence of cysts and spores. Fish with severe infection (> 10^5^) were anesthetized in buffered 3-aminobenzoic acid ethyl ether methanesulfonate (Sigma-Aldrich, St. Louis, MO, USA). Purified spores were diluted in Dulbecco’s Modified Eagle’s Medium (DMEM) (Life Technologies, Grand Island, NY, USA) for treatment of cultured Caco-2 cells.

### Cell culture

Caco-2 cells, a human intestine epithelial adenocarcinoma cell line, were purchased from the American Type Cell Culture (HTB37; Manassas, VA, USA). Caco-2 cells at passage 7 were maintained in DMEM (Life Technologies) supplemented with 20% fetal bovine serum, antibiotics (Life Technologies), and 1% non-essential amino acids (Life Technologies) at 37 °C in a 5% CO_2_ incubator. The medium was replaced twice a week and cell splitting done using 0.25% trypsin-EDTA (Life Technologies) upon 90% confluency (about 7–10 days post-seeding).

Purified *K*. *septempunctata* spores (equivalent to 2.5 × 10^6^ spores/well) were divided into naïve or heated spores and heated at 95 °C for 5 min. Each group of spores with or without denaturation was exposed to Caco-2 cells. The number of spores in each well was 2.5-fold that of the cell number for adherence to Caco-2 cells in a previous report [16]. Lipopolysaccharide (LPS; 100 μg/mL) (Sigma-Aldrich), a main component of the outer membrane of Gram-negative bacteria and one of the most potent stimuli of inflammation in the gut [20], served as the positive control.

Cells were seeded onto 6-cm cell culture dishes at a density of 1 × 10^6^ cells and incubated for 24 h. Next, the medium was changed to DMEM containing 1% non-essential amino acids, and cells were incubated with different statuses of *K*. *septempunctata* spores for 24 h at 37 °C in a 5% CO_2_ incubator. The infected cells were sampled for cytotoxicity using the lactate dehydrogenase (LDH) assay and western blot analysis, which was performed to examine the expression of inducible nitric oxide synthase (iNOS) and cyclooxygenase (COX)-2, and supernatants were collected for the analysis of nitric oxide (NO) and prostaglandin E2 (PGE_2_).

### LDH assay

To determine the cytotoxicity of *K*. *septempunctata* spores, LDH activity was measured using a Cytotoxicity Detection Kit (Dojindo Molecular Technologies, Rockville, MD, USA). To confirm the denaturation of *K*. *septempunctata* spores, we measured LDH release with and without lysis buffer (supplied in the kit). Next, Caco-2 cells were seeded onto 96-well plates at a density of 1 × 10^4^ cells/100 µL and grown to 80–90% confluence overnight at 37 °C in a 5% CO_2_ incubator. Cells were exposed to different statuses of *K*. *septempunctata* spores (2.5 × 10^4^ spores/dish) for 24 h. The LDH release assay was then performed according to the manufacturer’s protocol. Briefly, 100 µL of supernatant were transferred from each well to a 96-well plate and 100 µL of freshly prepared reaction mixture was added. After 30 min of incubation at 37 °C, 50 µL of stop solution were added, and absorbance was measured at 490 nm on a Biotrak II Visible Plate Reader (Amersham Biosciences, Piscataway, NJ, USA). The amount of LDH is expressed as percent relative to the total amount of LDH present in cells treated with lysis buffer.

### NO assay

The nitrite that accumulated in the culture medium was measured as an indicator of NO production based on the Griess method. Briefly, 50 µL of cell culture medium was mixed with 50 µL of Griess reagent containing 0.2% naphthylenthylene diamine and 2% sulfanilamide in 10% phosphoric acid for 10 min at 37 °C, and the absorbance was measured at 540 nm on a microplate reader (Amersham Biosciences). To generate the standard curve, a serial dilution of sodium nitrite was used.

### Measurement of PGE_2_

The culture supernatant was collected. The PGE_2_ level was measured using a PGE_2_ enzyme-linked immunosorbent assay kit, according to the manufacturer’s instructions (Abcam, Cambridge, MA, USA). Briefly, the diluted cell supernatant (100 µL) was placed on a goat anti-mouse IgG-coated 96-well plate and incubated for 2 h. After washing, the color was developed by adding p-nitrophenyl phosphate (200 µL) substrate after 45 min. The amount of PGE_2_ was calculated using a PGE_2_ standard curve.

### Western blotting

For protein preparation, Caco-2 cells were lysed in PRO-PREP^TM^ buffer (iNtRON Biotechnology, Kirkland, WA, USA). For the immunoblot assay, supernatants containing 40 µg of protein were loaded into individual lanes of an 8% sodium dodecyl sulfate-polyacrylamide gel, electrophoresed, and immunoblotted onto a nitrocellulose membrane (Schleicher and Schuell, Keene, NH, USA). The membrane was then incubated with mouse monoclonal anti-COX-2 (1:1,000 dilution; Santa Cruz Biotechnology, Dallas, TX, USA) or rabbit polyclonal anti-iNOS (1:1,000 dilution; Abcam) for 2 h. Bound antibodies were detected using a chemiluminescent substrate (Miracle-Star^TM^; iNtRON Biotech, Gyeonggi, Korea), according to the manufacturer’s instructions. After imaging, the membranes were stripped and reprobed with mouse monoclonal anti-β-actin (1:10,000 dilution; Sigma-Aldrich). The optical density (OD; per mm^2^) of each band was measured, and the density of the band relative to the density of that of β-actin was compared using ImageJ software (NIH, Bethesda, MD, USA).

### Statistical analysis

Data are presented as the mean ± standard error. Data were subjected to one-way analysis of variance followed by the Student–Newman–Keuls *post hoc* test for multiple comparisons. In all cases, *p* < 0.05 was considered to indicate significance.

## Results

**Figure 1:**
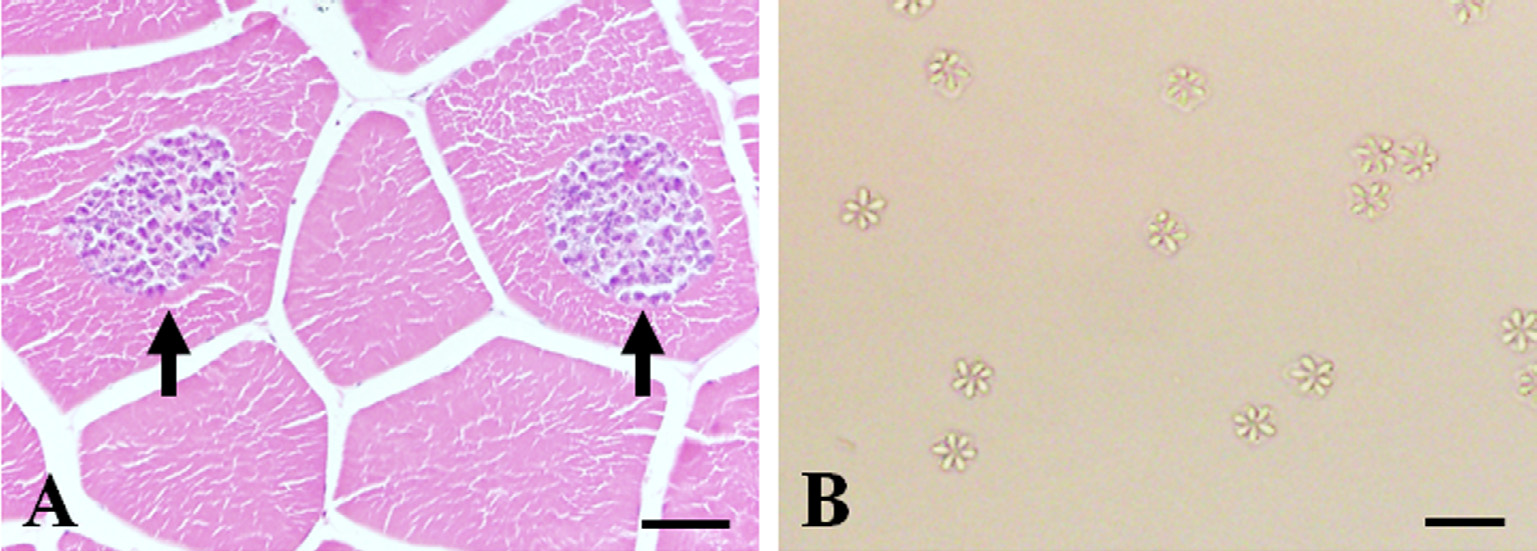
(A) Histology of olive flounder (*Paralichthys olivaceus*) muscles showing muscle fibers containing *Kudoa septempunctata* spores. Arrows indicate spore-containing cysts. Hematoxylin and eosin staining. (B) Microscopy of isolated *K*. *septempunctata* spores from olive flounder muscles. Scale bars: A, 100 µm; B, 10 µm.

*K*. *septempunctata* spores were isolated from infected olive flounder muscle from a fish farm in Jeju Island (Figure 1A, arrows). The spores contained six to seven shell valves and polar capsules per spore, consistent with previous reports (Figure 1B) [1,9].

**Figure 2:**
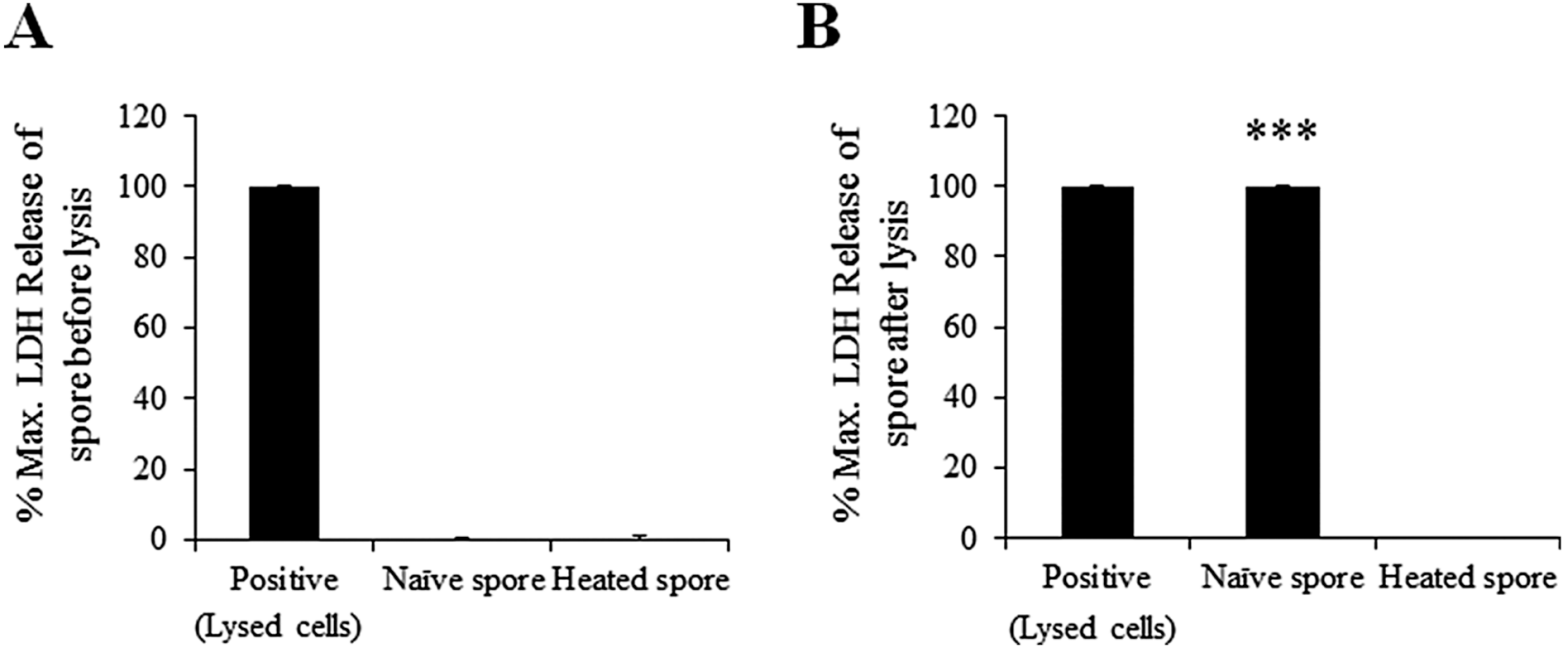
Cytotoxicity in naïve and heated spores of *Kudoa septempunctata*. Lactate dehydrogenase (LDH) release was measured before and after lysis. Data are presented as mean ± standard error (SE) of three independent experiments performed in triplicate. ***p < 0.001 vs. naïve spores before lysis.

The LDH assay was performed to examine cell membrane damage [11]. Neither naïve nor heated spores released LDH before lysis (Figure 2A). However, following lysis (using lysis buffer contained in the kit), LDH release increased by 100% in naïve spores, but remained unchanged in heated spores (Figure 2B). These results show that heating denatured *K*. *septempunctata* spores, consistent with a previous report [21].

**Figure 3:**
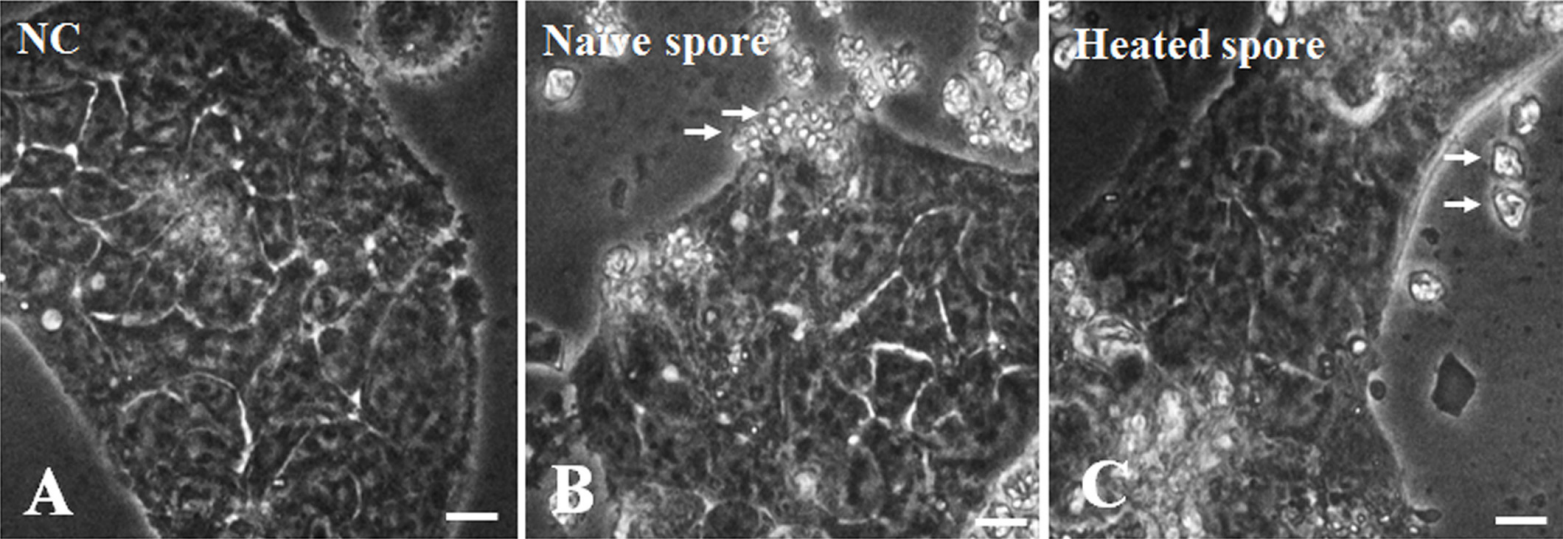
Microscopy of naïve and heated spores of *Kudoa septempunctata* in Caco-2 cells. (A) Vehicle-treated negative control, (B) naïve spores (arrows), and heated spores (arrows) exposed to Caco-2 cells. Scale bars, 50 µm.

These conditional spores were exposed to Caco-2 cells and, upon examination, were located near the Caco-2 cells. When cells are exposed to naïve or heated spores (Figure 3B and C), they develop the morphologic characteristics of normal enterocytes when grown on plastic dishes [7] (Figure 3A–C). Naïve spores contained six to seven shell valves and polar capsules per spore (Figure 3B, arrows), but heated spores appeared as wrinkled shell valves and polar capsules (Figure 3C, arrows).

**Figure 4:**
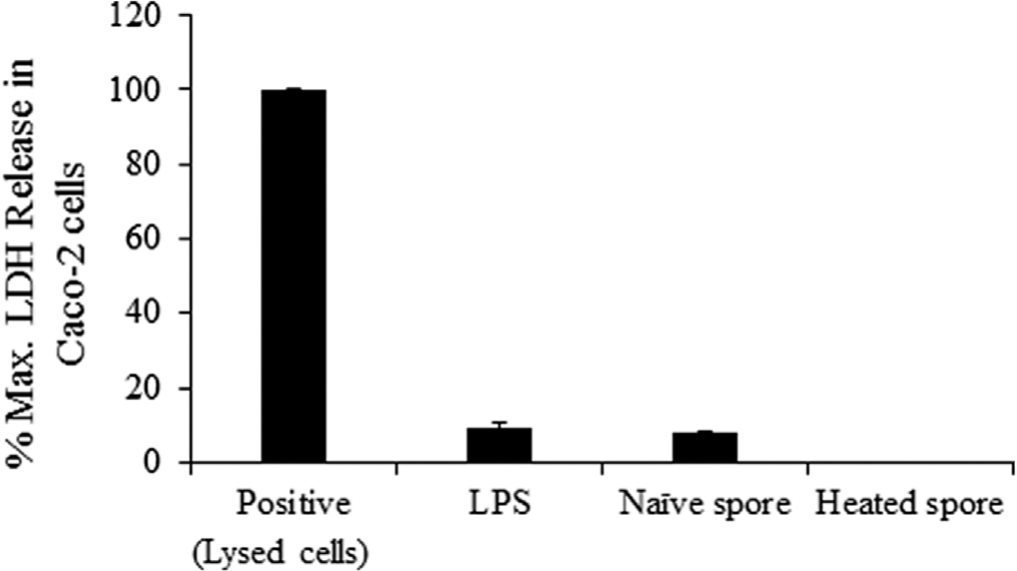
Analysis of cytotoxicity in Caco-2 cells following exposure to naïve and heated spores of *Kudoa septempunctata*. Cytotoxicity was measured based on LDH release. Data are presented as mean ± SE of three independent experiments performed in triplicate.

The mean cytotoxicity of the LPS positive control (100 μg/mL) was 100%, and that of naïve and heated spores was under 10% (Figure 4). Therefore, we selected this concentration for subsequent experiments.

**Figure 5:**
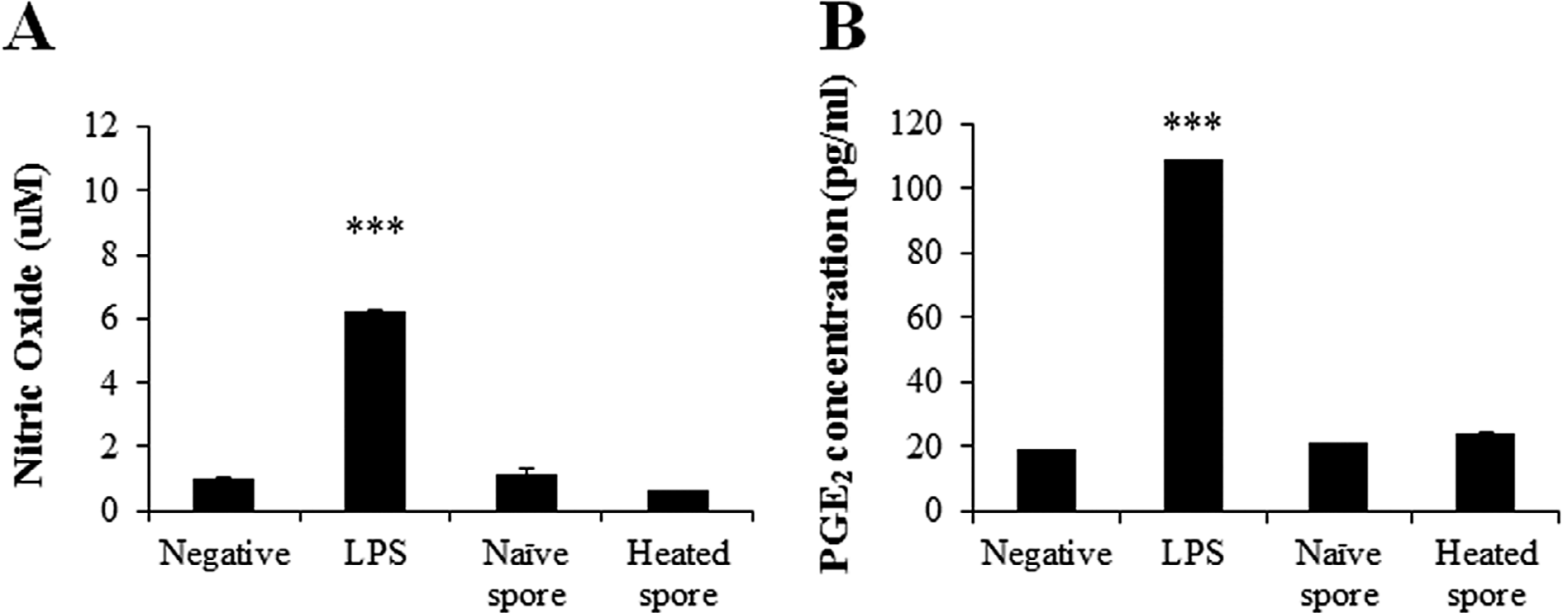
Effects of *Kudoa septempunctata* spores on nitric oxide (NO) (A) and prostaglandin E2 (PGE_2_) (B) production in Caco-2 cells. Data are presented as mean ± SE of three independent experiments performed in triplicate. ***p < 0.001 vs. negative control.

To analyze the inflammatory properties of *K*. *septempunctata*, we measured the levels of NO and PGE_2_ upon exposure to naïve and heated spores. Additionally, both LPS and vehicle were added, which served as the positive and negative control, respectively. After the cell culture media was collected, NO and PGE_2_ levels were determined, and both naïve and heated spores exhibited changes in NO and PGE_2_ production compared with the negative control (Figure 5A). Additionally, LPS-stimulated cells showed significantly increased production of NO and PGE_2_ (relative OD values, 6.25 ± 0.17-fold, *p* < 0.001 and 5.74 ± 0.03-fold, *p* < 0.001, respectively) compared with those of the negative control. However, their production remained unchanged in both naïve and heated spores (Figure 5B).

**Figure 6:**
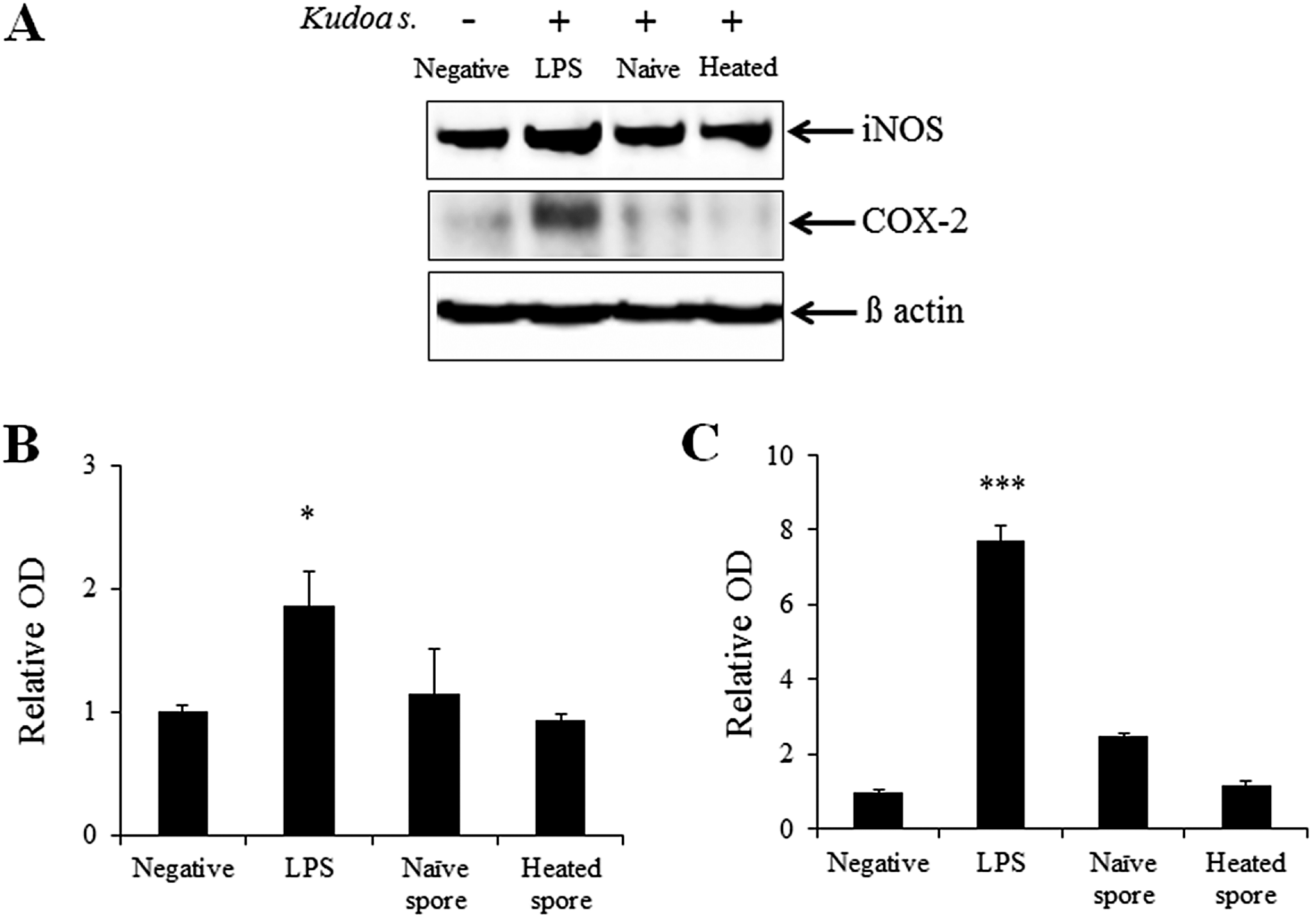
Western blot analysis of inducible nitric oxide synthase (iNOS) and cyclooxygenase (COX)-2 expression in Caco-2 cells with or without spores of *Kudoa septempunctata*. (A) Representative immunoblots of iNOS (∼120 kDa), COX-2 (∼72 kDa), and β-actin (∼45 kDa) expression. (B and C) Bar graphs show a significant increase in iNOS (B) and COX-2 (C) expression in LPS-exposed Caco-2 cells, and no change following exposure to *K*. *septempunctata* spores. For normalization, membranes were reprobed with anti-β-actin. Data are presented as mean ± SE of three independent experiments performed in triplicate. *p < 0.05, ***p < 0.001 vs. negative control.

Western blot analysis was performed to examine the expression of iNOS and COX-2, which are related to the modulation of the expression of NO and PGE_2_. The protein levels of iNOS and COX-2 in Caco-2 cells were lower than the negative control. The expression of iNOS and COX-2 proteins was significantly increased (relative OD values, 1.87 ± 0.26-fold, *p* < 0.05 and 7.70 ± 0.43-fold, *p* < 0.001, respectively) in LPS-exposed Caco-2 cells compared with the negative control (Figure 6A–C). In contrast, the exposed groups of *K*. *septempunctata* spores showed a change in the protein level of iNOS or COX-2, similar to the negative control (Figure 6A–C).

## Discussion

This report provides the first confirmation of inflammatory responses in *K*. *septempunctata* spore-exposed Caco-2 cells. In addition, we confirmed that *K*. *septempunctata* spores can be denatured by heating. Because the release of LDH by heated spores did not change following lysis, we hypothesized that the cell membranes of the heated spores were not yet damaged. Our results indicate that LDH release remained unchanged after lysis with lysis buffer. Therefore, we conclude that the *K*. *septempunctata* spores were denatured upon heating at 95 °C for 5 min. These results are in accordance with those of a previous report showing that *K*. *septempunctata* spores could be eradicated by heating at temperatures greater than 80 °C [21]. Moreover, based on the calculated viability of the damaged spores, it is likely that treatments such as heating, ethanol, and chemical stimulation can effectively kill *K*. *septempunctata* spores [21].

The Caco-2 cell line is the most widely used epithelial cell monolayer for drug transport studies [17] and a target of common causative agents of bacterial food poisoning, including *Clostridium perfringens* enterotoxin [6] and enterohemorrhagic *Escherichia coli* [3]. Many species of protozoa can induce diarrhea through the invasion of, and reproduction in, the intestine [5]. However, although *K*. *septempunctata* is able to invade the intestine [16], it has not been reported whether *K*. *septempunctata* can reproduce there and cause diarrhea. Therefore, we examined whether exposure to *K*. *septempunctata* spores is cytotoxic to Caco-2 cells and could induce the secretion of inflammatory mediators. In a previous study, the invasion of Caco-2 cells by naïve sporoplasms of *K*. *septempunctata* occurred relatively rapidly, resulting in the termination of monolayer confluence, as illustrated by a rapid loss of transepithelial electrical resistance as an indicator of permeability due to the severe damage of individual cells within the monolayer [16]. In contrast to that study, our results show that *K*. *septempunctata* spores did not damage Caco-2 cells by releasing LDH. It has been reported that *K*. *septempunctata* can cause disease in humans [16], but the mechanism whereby it causes inflammation in the human intestine has not been established.

We investigated whether *K*. *septempunctata* spores induce inflammatory mediators in the Caco-2 human intestinal epithelial cell line. In this study, we exposed naïve and heated spores to Caco-2 cells and confirmed whether the addition of spores enhanced the secretion of NO and PGE_2_ and related proteins, including iNOS and COX-2. *K*. *septempunctata* spores in Caco-2 cells did not induce the secretion of inflammatory mediators, contrary to LPS stimulation (positive control). Inflammation is one of the most crucial aspects of the host defense against invading pathogens [2]. During the inflammatory process, inflammatory mediators such as NO and PGE_2_ are generated by iNOS and COX-2 [8]. However, in this study, these mediators (NO, PGE_2_, iNOS, and COX-2) remained unchanged in Caco-2 cells following exposure to *K*. *septempunctata* spores (both naïve and heated). We believe that naïve and heated spores of *K*. *septempunctata* are not associated with inflammation (leading to diarrhea) in Caco-2 cells. However, the pathogenicity of *K*. *septempunctata* spores should be studied further to analyze pro-inflammatory cytokines.

Taken together, the results of this study suggest that *K*. *septempunctata* spores are denatured by heating (as evidenced by the LDH assay) and are not cytotoxic to Caco-2 cells, a human intestinal epithelial cell line. Moreover, *K*. *septempunctata* spores did not affect the expression of inflammatory mediators, including NO, PGE_2_, iNOS, and COX-2, in Caco-2 cells. This study implies that spores of *K*. *septempunctata* genotype ST3 do not cause intestinal inflammation *in vitro*.

## Conflict of interest

The authors declare that they have no conflict of interest.
